# Supplementary material for: MOGONET integrates multi-omics data using graph convolutional networks allowing patient classification and biomarker identification
Source: Nat Commun. 2021 Jun 8;12:3445. doi: 10.1038/s41467-021-23774-w (PMC8187432; doi:10.1038/s41467-021-23774-w)
Supplement: Supplementary file 1 — Supplementary Information [file 41467_2021_23774_MOESM1_ESM.pdf]

Supplementary information of

MOGONET integrates multi-omics data using graph convolutional networks allowing patient classification and biomarker identification

Supplementary Tables

Table S1. Classification results on KIPAN dataset

Method	ACC	F1_weighted	F1_macro
KNN	0.967 ± 0.011	0.967 ± 0.011	0.960 ± 0.014
SVM	0.995 ± 0.003	0.995 ± 0.003	0.994 ± 0.004
Lasso	0.974 ± 0.002	0.974 ± 0.002	0.972 ± 0.004
RF	0.981 ± 0.006	0.981 ± 0.006	0.975 ± 0.011
XGBoost	0.993 ± 0.008	0.993 ± 0.008	0.989 ± 0.014
NN	0.991 ± 0.005	0.991 ± 0.005	0.991 ± 0.005
GRridge	0.994 ± 0.004	0.994 ± 0.004	0.993 ± 0.004
block PLSDA	0.933 ± 0.013	0.933 ± 0.013	0.919 ± 0.021
block sPLSDA	0.919 ± 0.012	0.918 ± 0.013	0.895 ± 0.014
NN_NN	0.988 ± 0.011	0.988 ± 0.011	0.981 ± 0.016
NN_VCDN	0.991 ± 0.005	0.991 ± 0.005	0.991 ± 0.005
MOGONET_NN (Ours)	0.997 ± 0.004	0.997 ± 0.004	0.998 ± 0.003
MOGONET (Ours)	0.999 ± 0.002	0.999 ± 0.002	0.999 ± 0.002

Table S2. Classification results on BRCA dataset (Normal-like vs. Non-normal-like)

Method	ACC	F1	AUC
KNN	0.837 ± 0.012	0.909 ± 0.008	0.789 ± 0.029
SVM	0.830 ± 0.014	0.906 ± 0.009	0.525 ± 0.026
Lasso	0.866 ± 0.002	0.928 ± 0.001	0.928 ± 0.018
RF	0.833 ± 0.011	0.908 ± 0.006	0.898 ± 0.011
XGBoost	0.840 ± 0.009	0.910 ± 0.005	0.901 ± 0.006
NN	0.837 ± 0.015	0.909 ± 0.008	0.895 ± 0.020
GRridge	0.832 ± 0.015	0.906 ± 0.009	0.887 ± 0.017
block PLSDA	0.867 ± 0.000	0.929 ± 0.000	0.939 ± 0.017
block sPLSDA	0.867 ± 0.000	0.929 ± 0.000	0.937 ± 0.018
NN_NN	0.852 ± 0.016	0.917 ± 0.009	0.917 ± 0.015
NN_VCDN	0.852 ± 0.014	0.916 ± 0.008	0.909 ± 0.017
MOGONET_NN (Ours)	0.864 ± 0.009	0.926 ± 0.005	0.915 ± 0.012
MOGONET (Ours)	0.869 ± 0.010	0.927 ± 0.005	0.933 ± 0.015

Table S3. Classification results on BRCA dataset (Normal-like vs. Basal-like vs. HER2-enriched vs. Luminal (LumA and LumB))

Method	ACC	F1_weighted	F1_macro
KNN	0.834 ± 0.015	0.825 ± 0.015	0.718 ± 0.020
SVM	0.814 ± 0.015	0.788 ± 0.013	0.670 ± 0.016
Lasso	0.821 ± 0.013	0.781 ± 0.022	0.610 ± 0.056
RF	0.800 ± 0.010	0.769 ± 0.009	0.588 ± 0.019
XGBoost	0.827 ± 0.009	0.810 ± 0.012	0.698 ± 0.024
NN	0.818 ± 0.018	0.801 ± 0.024	0.673 ± 0.048
GRridge	0.814 ± 0.014	0.794 ± 0.014	0.668 ± 0.025
block PLSDA	0.807 ± 0.009	0.737 ± 0.012	0.476 ± 0.014
block sPLSDA	0.805 ± 0.009	0.731 ± 0.010	0.467 ± 0.011
NN_NN	0.833 ± 0.009	0.815 ± 0.009	0.705 ± 0.020
NN_VCDN	0.837 ± 0.006	0.823 ± 0.009	0.715 ± 0.022
MOGONET_NN (Ours)	0.840 ± 0.008	0.800 ± 0.018	0.692 ± 0.044
MOGONET (Ours)	0.859 ± 0.018	0.845 ± 0.023	0.747 ± 0.032

Table S4. Classification results of MOGONET on BRCA dataset by training and testing on samples from different institutions

Labels	Evaluation metrics		
Normal-like, Basal-like, HER2-enriched, Luminal A, Luminal B	ACC	F1_weighted	F1_macro
	0.829	0.830	0.781
Normal-like, Non-normal-like	ACC	F1	AUC
	0.886	0.936	0.923
Normal-like, Basal-like, HER2-enriched, Luminal	ACC	F1_weighted	F1_macro
	0.869	0.868	0.778

Training samples are from institutions with Tissue Source Side Code of 5L, UL, GM, EW, LD, AR, A2, V7, 4H, 5T, 3C, XX, Z7, LQ, LL, UU, AQ, WT, AN, W8, S3, MS, GI, A7, D8, HN, BH, and B6 (577 samples). Test samples are from institutions with Tissue Source Side Code of A1, A8, AC, AO, C8, E2, E9, JL, OK, OL, PE, and PL (298 samples).

Table S5. Classification results on BRCA dataset with mRNA expression and DNA methylation data

Method	ACC	F1_weighted	F1_macro
KNN	0.736 ± 0.022	0.720 ± 0.022	0.670 ± 0.031
SVM	0.738 ± 0.024	0.722 ± 0.025	0.664 ± 0.023
Lasso	0.729 ± 0.011	0.695 ± 0.011	0.637 ± 0.030
RF	0.757 ± 0.011	0.738 ± 0.016	0.664 ± 0.017
XGBoost	0.773 ± 0.010	0.757 ± 0.013	0.691 ± 0.019
NN	0.770 ± 0.017	0.757 ± 0.020	0.692 ± 0.028
GRridge	0.749 ± 0.020	0.730 ± 0.023	0.663 ± 0.027
block PLSDA	0.633 ± 0.007	0.511 ± 0.008	0.339 ± 0.013
block sPLSDA	0.633 ± 0.007	0.510 ± 0.009	0.336 ± 0.011
NN_NN	0.773 ± 0.048	0.765 ± 0.036	0.720 ± 0.026
NN_VCDN	0.788 ± 0.012	0.781 ± 0.011	0.714 ± 0.024
MOGONET_NN (Ours)	0.810 ± 0.015	0.790 ± 0.028	0.750 ± 0.031
MOGONET (Ours)	0.815 ± 0.019	0.804 ± 0.026	0.755 ± 0.031

Table S6. Classification results on BRCA dataset evaluated by AUC metrics

Method	AUC_weighted	AUC_macro
KNN	0.855 ± 0.011	0.844 ± 0.012
SVM	0.781 ± 0.014	0.774 ± 0.015
Lasso	0.923 ± 0.011	0.923 ± 0.010
RF	0.927 ± 0.006	0.917 ± 0.007
XGBoost	0.943 ± 0.007	0.932 ± 0.010
NN	0.945 ± 0.009	0.937 ± 0.012
GRridge	0.936 ± 0.008	0.929 ± 0.009
block PLSDA	0.895 ± 0.008	0.889 ± 0.010
block sPLSDA	0.894 ± 0.010	0.888 ± 0.013
NN_NN	0.957 ± 0.007	0.950 ± 0.009
NN_VCDN	0.948 ± 0.009	0.939 ± 0.013
MOGONET_NN (Ours)	0.955 ± 0.003	0.949 ± 0.005
MOGONET (Ours)	0.960 ± 0.005	0.952 ± 0.006

Table S7. Classification results on KIPAN dataset evaluated by AUC metrics

Method	AUC_weighted	AUC_macro
KNN	0.987 ± 0.004	0.987 ± 0.004
SVM	0.994 ± 0.004	0.993 ± 0.005
Lasso	0.993 ± 0.007	0.994 ± 0.006
RF	0.999 ± 0.001	0.999 ± 0.001
XGBoost	0.998 ± 0.002	0.998 ± 0.002
NN	0.999 ± 0.000	0.999 ± 0.001
GRridge	1.000 ± 0.000	1.000 ± 0.000
block PLSDA	0.987 ± 0.003	0.987 ± 0.003
block sPLSDA	0.983 ± 0.003	0.984 ± 0.004
NN_NN	0.999 ± 0.001	0.999 ± 0.001
NN_VCDN	0.997 ± 0.003	0.997 ± 0.003
MOGONET_NN (Ours)	0.999 ± 0.001	0.999 ± 0.001
MOGONET (Ours)	1.000 ± 0.001	1.000 ± 0.001

Table S8. Important omics biomarkers identified by MOGONET in the LGG dataset

Omics data type	Biomarkers
mRNA expression (14)	*DGKK*, *PRKG2*, *IGFN1*, *TMEM179*, *DACH2*, *SFTPD*, *CSMD1*, *PON1*, *C11orf20*, *LRFN5*, *PCSK6*, *TMEM196*, *MUM1L1*, *LOC349196*
DNA methylation (9)	*OR6Q1*, *SIGLEC11*, *DEFB126*, *LOC553137*, *MYOZ2*, *SPA17*, *CST5*, *SPRR2D*, *MIR150*
miRNA expression (7)	*hsa-mir-183*, *hsa-mir-383*, *hsa-mir-184*, *hsa-mir-204*, *hsa-mir-152*, *hsa-mir-148a*, *hsa-mir-219-2*

Table S9. Ranking of omics biomarkers identified by MOGONET in the ROSMAP dataset

Rank	Biomarkers	Omics data type
1	*hsa-miR-423-3p*	miRNA expression
2	*hsa-miR-33a*	miRNA expression
3	*hsa-miR-640*	miRNA expression
4	*hsa-miR-362-3p*	miRNA expression
5	*hsa-miR-491-5p*	miRNA expression
6	*NPNT*	mRNA expression
7	*TMC4*	DNA methylation
8	*CDK18*	mRNA expression
9	*AGA*	DNA methylation
10	*hsa-miR-206*	miRNA expression
11	*KIF5A*	mRNA expression
12	*hsa-miR-548b-3p*	miRNA expression
13	*hsa-miR-127-3p*	miRNA expression
14	*hsa-miR-106a_hsa-miR-17*	miRNA expression
15	*HYAL2*	DNA methylation
16	*SPACA6*	mRNA expression
17	*hsa-miR-424*	miRNA expression
18	*hsa-miR-577*	miRNA expression
19	*hsa-miR-873*	miRNA expression
20	*CCL3*	DNA methylation
21	*TCEA3*	mRNA expression
22	*SYTL1*	mRNA expression
23	*hsa-miR-651*	miRNA expression
24	*hsa-miR-199b-5p*	miRNA expression
25	*hsa-miR-192*	miRNA expression
26	*TTC15*	DNA methylation
27	*hsa-miR-199a-5p*	miRNA expression
28	*ARRDC2*	mRNA expression
29	*hsv1-miR-H1*	miRNA expression
30	*APLN*	mRNA expression

Table S10. Ranking of omics biomarkers identified by MOGONET in the BRCA dataset

Rank	Biomarkers	Omics data type
1	*SOX11*	mRNA expression
2	*hsa-mir-205*	miRNA expression
3	*GPR37L1*	DNA methylation
4	*AMY1A*	mRNA expression
5	*SLC6A15*	mRNA expression
6	*FABP7*	mRNA expression
7	*MIR563*	DNA methylation
8	*SLC6A14*	mRNA expression
9	*hsa-mir-187*	miRNA expression
10	*SLC6A2*	mRNA expression
11	*FGFBP1*	mRNA expression
12	*DSG1*	mRNA expression
13	*UGT8*	mRNA expression
14	*ANKRD45*	mRNA expression
15	*OR1J4*	DNA methylation
16	*ATP10B*	DNA methylation
17	*PI3*	mRNA expression
18	*hsa-mir-452*	miRNA expression
19	*hsa-mir-20b*	miRNA expression
20	*SERPINB5*	mRNA expression
21	*KRTAP3-3*	DNA methylation
22	*COL11A2*	mRNA expression
23	*hsa-mir-224*	miRNA expression
24	*FLJ41941*	DNA methylation
25	*ARHGEF4*	mRNA expression
26	*SOX10*	mRNA expression
27	*TMEM207*	DNA methylation
28	*CDH26*	DNA methylation
29	*MT1DP*	DNA methylation
30	*hsa-mir-204*	miRNA expression

Table S11. Ranking of omics biomarkers identified by MOGONET in the LGG dataset

Rank	Biomarkers	Omics data type
1	*DGKK*	mRNA expression
2	*OR6Q1*	DNA methylation
3	*PRKG2*	mRNA expression
4	*SIGLEC11*	DNA methylation
5	*hsa-mir-183*	miRNA expression
6	*DEFB126*	DNA methylation
7	*IGFN1*	mRNA expression
8	*hsa-mir-383*	miRNA expression
9	*hsa-mir-184*	miRNA expression
10	*TMEM179*	mRNA expression
11	*LOC553137*	DNA methylation
12	*hsa-mir-204*	miRNA expression
13	*MYOZ2*	DNA methylation
14	*SPA17*	DNA methylation
15	*hsa-mir-152*	miRNA expression
16	*DACH2*	mRNA expression
17	*hsa-mir-148a*	miRNA expression
18	*SFTPD*	mRNA expression
19	*CSMD1*	mRNA expression
20	*PON1*	mRNA expression
21	*hsa-mir-219-2*	miRNA expression
22	*CST5*	DNA methylation
23	*SPRR2D*	DNA methylation
24	*C11orf20*	mRNA expression
25	*LRFN5*	mRNA expression
26	*PCSK6*	mRNA expression
27	*MIR150*	DNA methylation
28	*TMEM196*	mRNA expression
29	*MUM1L1*	mRNA expression
30	*LOC349196*	mRNA expression

Table S12. Important omics biomarkers identified by sPLSDA in the ROSMAP dataset

Omics data type	Biomarkers
mRNA expression (28)	*CARHSP1*, *SLC6A12*, *QDPR*, *NDE1*, *AQP6*, *CSRP1*, *APLN*, *MCM7*, *ANKRD40*, *GAREM2*, *PRELP*, *PRTN3*, *ANLN*, *KIF5B*, *S100A4*, *CCDC69*, *PPDPF*, *SLC43A3*, *SLC6A9*, *PLEKHB1*, *PREX1*, *CPM*, *ARRDC2*, *MID1IP1*, *SLC4A11*, *CDK18*, *FAM107B*, *CDK2AP1*
DNA methylation (0)	
miRNA expression (2)	*hsa-miR-129-5p*, *hsa-miR-132*

Table S13. Important omics biomarkers identified by sPLSDA in the BRCA dataset

Omics data type	Biomarkers
mRNA expression (20)	*ABCC11*, *ACE2*, *ANP32E*, *AR*, *C9orf152*, *CAPN13*, *CCKBR*, *CYP4Z2P*, *FAM19A3*, *FOXC1*, *GDF5*, *ISL2*, *MFI2*, *MPHOSPH6*, *PRTFDC1*, *SEC16A*, *SIDT1*, *SOX11*, *SPDEF*, *TMC5*
DNA methylation (7)	*IGFBP4*, *NPSR1*, *RAPGEF1*, *SCUBE2*, *SIK1*, *TKTL2*, *ZNF516*
miRNA expression (3)	*hsa-mir-2115*, *hsa-mir-30a*, *hsa-mir-30c-2*

Table S14. Important omics biomarkers identified by sPLSDA in the LGG dataset

Omics data type	Biomarkers
mRNA expression (30)	*ATF3*, *C11orf21*, *CALHM2*, *CXorf36*, *ECE1*, *F11R*, *FBXO22OS*, *FOSL1*, *FOXC1*, *GCLC*, *HBEGF*, *HEXB*, *HLA-DPB1*, *HSPA7*, *IRF1*, *ISG20*, *ITGA5*, *KCND2*, *KLHL25*, *LRRN4CL*, *LSP1*, *NAB2*, *PLK3*, *PRSS35*, *SERPINA3*, *SERPINA5*, *SERPING1*, *SOCS3*, *TRIM21*, *VEPH1*
DNA methylation (0)	
miRNA expression (0)	

Supplementary Figures

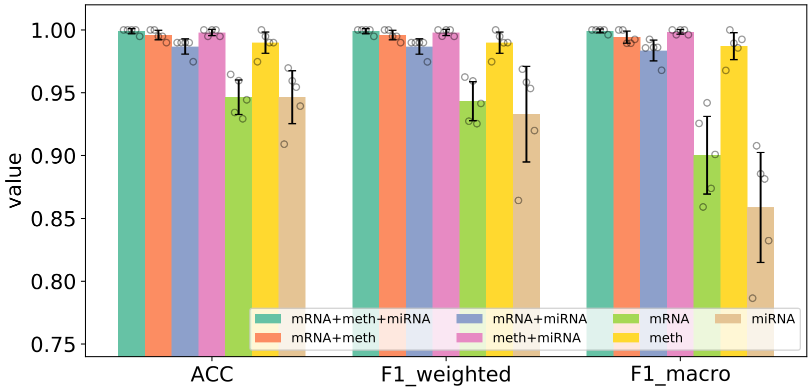

Figure S1. Performance comparison of multi-omics data classification via MOGONET and single-omics data classification via GCN on the KIPAN dataset ($n = 5$ experiments for each model). Means of evaluation metrics with standard deviations from different experiments are shown in the figure, where the error bar represents plus/minus one standard deviation. mRNA, meth, and miRNA refer to single-omics data classification via GCN with mRNA expression data, DNA methylation data, and miRNA expression data, respectively. mRNA+meth, mRNA+miRNA, and meth+miRNA refer to classification with two types of omics data. mRNA+meth+miRNA refers to classification with three types of omics data. Source data are provided as a Source Data file.

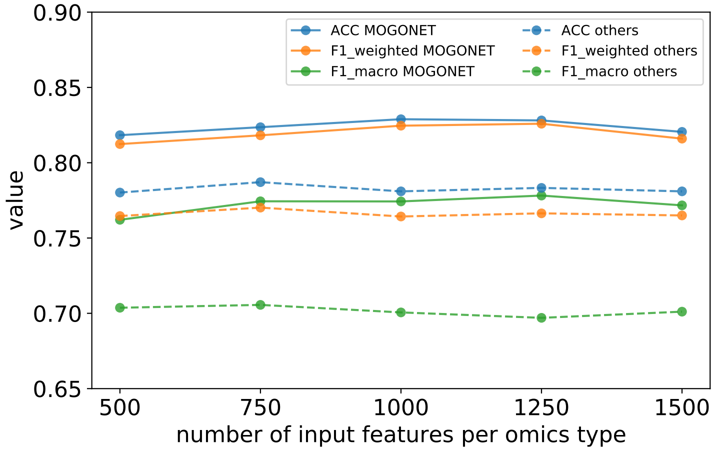

Figure S2. Performance of MOGONET under different numbers of input omics features on the BRCA dataset. MOGONET produced similar results under a wide range of numbers of input features and consistently outperformed the best existing methods (XGBoost) in the BRCA dataset. For the number of input features per omics type in the x-axis, if the number of input features was larger than the total number of features for an omics data type, all the features from that omics data type were used for training. Source data are provided as a Source Data file.

Supplementary Discussion

MOGONET identified biomarkers related to glioma

For LGG grade classification, 14 mRNA features, nine DNA methylation features, and seven miRNA features were identified by MOGONET as the top 30 important biomarkers (Supplementary Table 8). For genes identified by mRNA expression features, GO terms related to *PRKG2* like cGMP-dependent protein kinase activity (GO:0004692, $p = 3.24E-2$) were significantly enriched. Swartling et al.[1] demonstrated that the expression of cyclic guanosine monophosphate (cGMP)-dependent protein kinase II, cGKII, in glioma cells in culture provided a growth inhibitory signal, where cGKII was encoded by *PRKG2*. For genes related to the identified DNA methylation features by MOGONET, molecular functions like sialic acid binding (GO:0033691, $p = 4.50E-2$) were significantly enriched. Santegoets et al.[2] suggested possible sialic acid–Siglec interactions between glioma cells and myeloid-derived suppressor cells (MDSCs) in the tumor microenvironment, which could contribute to the immunosuppressive role of MDSCs in glioma patients. On the other hand, for biomarkers identified by sPLSDA, biological process terms such as negative regulation of protein metabolic process (GO:0051248, $p = 7.93E-3$) and regulation of cell death (GO:0010941, $p = 4.92E-2$) were significantly enriched, which have been shown to play important roles in the proliferative drive of cancers[3,4].

Moreover, highly-ranked genes and miRNAs identified by MOGONET have also been shown to be associated with glioma. For example, besides *PRKG2* mentioned above, *SIGLEC11* mRNA expression has been shown to be downregulated in LGG[5]. Chen et al.[5] also demonstrated that most of the Siglec family genes were correlated with CD4+ T cell while negatively correlated with CD8+ T cell infiltration in LGG. Besides, Auvergne et al.[6] suggested a critical role of the *SIX1-EYA1-DACH2* system in gliomagenesis or glioma progression. Delic et al.[7] identified and validated *PCSK6* as a glioma invasion-associated candidate gene where the knockdown of *PCSK6* inhibited glioma cell invasion. For the identified miRNAs, *miRNA-183* has been previously shown to be a contributor to glioma progression through regulation of the Pax6-5a isoform[8]. Elevated expression levels of the *miRNA-183* cluster members have also been observed in gliomas from multiple studies[9–11]. Moreover, Xu et al.[12] found that the expression level of *miRNA-383* was negatively related to the malignant degree of gliomas and further demonstrated that *miRNA-383* played the role of tumor suppressor in glioma cells by downregulating *CCND1* expression. He et al.[13] also revealed a novel regulatory mechanism in glioma, where the downregulation of *miRNA-383* could contribute to *IGF1R* signaling and glioma cell invasion.

References

1. Swartling, F. J., Ferletta, M., Kastemar, M., Weiss, W. & Westermark, B. Cyclic gmp-dependent protein kinase ii inhibits cell proliferation, sox9 expression and akt phosphorylation in human glioma cell lines. *Oncogene* **28**, 3121–3131 (2009).
2. Santegoets, K. C. *et al.* Expression profiling of immune inhibitory siglecs and their ligands in patients with glioma. *Cancer Immunol. Immunother.* **68**, 937–949 (2019).
3. Collins, K., Jacks, T. & Pavletich, N. P. The cell cycle and cancer. *Proc. Natl. Acad. Sci.* **94**, 2776–2778 (1997).
4. Vettore, L., Westbrook, R. L. & Tennant, D. A. New aspects of amino acid metabolism in cancer. *Br. journal cancer* **122**, 150–156 (2020).
5. Chen, Z. *et al.* Tumor derived siglec family genes may play roles in tumor genesis, progression, and immune microenvironment regulation. *Front. Oncol.* **10** (2020).
6. Auvergne, R. M. *et al.* Transcriptional differences between normal and glioma-derived glial progenitor cells identify a core set of dysregulated genes. *Cell reports* **3**, 2127–2141 (2013).
7. Delic, S., Lottmann, N., Jetschke, K., Reifenberger, G. & Riemenschneider, M. J. Identification and functional validation of cdh11, pcsk6 and sh3gl3 as novel glioma invasion-associated candidate genes. *Neuropathol. applied neurobiology* **38**, 201–212 (2012).
8. Pavlakis, E., Tonchev, A. B., Kaprelyan, A., Enchev, Y. & Stoykova, A. Interaction between transcription factors pax6/pax6-5a and specific members of mir-183-96-182 cluster, may contribute to glioma progression in glioblastoma cell lines. *Oncol. Reports* **37**, 1579–1592 (2017).
9. Yan, Z. *et al.* mir-96/hbp1/wnt/β-catenin regulatory circuitry promotes glioma growth. *FEBS letters* **588**, 3038–3046 (2014).
10. Song, L. *et al.* Tgf-β induces mir-182 to sustain nf-κb activation in glioma subsets. *The J. clinical investigation* **122**, 3563–3578 (2012).
11. Tanaka, H. *et al.* Microrna-183 upregulates hif-1α by targeting isocitrate dehydrogenase 2 (idh2) in glioma cells. *J. neuro-oncology* **111**, 273–283 (2013).
12. Xu, Z. *et al.* Microrna-383 inhibits anchorage-independent growth and induces cell cycle arrest of glioma cells by targeting ccnd1. *Biochem. biophysical research communications* **453**, 833–838 (2014).
13. He, Z. *et al.* Downregulation of mir-383 promotes glioma cell invasion by targeting insulin-like growth factor 1 receptor. *Med. Oncol.* **30**, 557 (2013).
