# Supplementary material for: MOGONET integrates multi-omics data using graph convolutional networks allowing patient classification and biomarker identification
Source: Nat Commun. 2021 Jun 8;12:3445. doi: 10.1038/s41467-021-23774-w (PMC8187432; doi:10.1038/s41467-021-23774-w)
Supplement: Supplementary file 3 — Reporting Summary [file 41467_2021_23774_MOESM3_ESM.pdf]

nature research

Corresponding author(s): Zhengming Ding and Kun Huang

Last updated by author(s): Apr 29, 2021

Reporting Summary

Nature Research wishes to improve the reproducibility of the work that we publish. This form provides structure for consistency and transparency in reporting. For further information on Nature Research policies, see our Editorial Policies and the Editorial Policy Checklist.

Statistics

For all statistical analyses, confirm that the following items are present in the figure legend, table legend, main text, or Methods section.

n/a	Confirmed	
☐	☒	The exact sample size (*n*) for each experimental group/condition, given as a discrete number and unit of measurement
☐	☒	A statement on whether measurements were taken from distinct samples or whether the same sample was measured repeatedly
☐	☒	The statistical test(s) used AND whether they are one- or two-sided<br>*Only common tests should be described solely by name; describe more complex techniques in the Methods section.*
☐	☒	A description of all covariates tested
☐	☒	A description of any assumptions or corrections, such as tests of normality and adjustment for multiple comparisons
☐	☒	A full description of the statistical parameters including central tendency (e.g. means) or other basic estimates (e.g. regression coefficient) AND variation (e.g. standard deviation) or associated estimates of uncertainty (e.g. confidence intervals)
☐	☒	For null hypothesis testing, the test statistic (e.g. *F*, *t*, *r*) with confidence intervals, effect sizes, degrees of freedom and *P* value noted<br>*Give P values as exact values whenever suitable.*
☒	☐	For Bayesian analysis, information on the choice of priors and Markov chain Monte Carlo settings
☒	☐	For hierarchical and complex designs, identification of the appropriate level for tests and full reporting of outcomes
☒	☐	Estimates of effect sizes (e.g. Cohen's *d*, Pearson's *r*), indicating how they were calculated

Our web collection on statistics for biologists contains articles on many of the points above.

Software and code

Policy information about availability of computer code

Data collection	PAM50 breast cancer subtypes of TCGA BRCA patients were obtained through the TCGAbiolinks R package (v2.12.6, http://bioconductor.org/packages/release/bioc/html/TCGAbiolinks.html).
Data analysis	The source code of MOGONET can be downloaded from GitHub (https://github.com/txWang/MOGONET). XGBoost python package (v0.90), scikit-learn python package (v0.23.2), and mixOmics R package (v6.10.9) were also used for data analysis.

For manuscripts utilizing custom algorithms or software that are central to the research but not yet described in published literature, software must be made available to editors and reviewers. We strongly encourage code deposition in a community repository (e.g. GitHub). See the Nature Research guidelines for submitting code & software for further information.

Data

Policy information about availability of data

All manuscripts must include a data availability statement. This statement should provide the following information, where applicable:
- Accession codes, unique identifiers, or web links for publicly available datasets
- A list of figures that have associated raw data
- A description of any restrictions on data availability

The ROSMAP dataset was obtained from AMP-AD Knowledge Portal (https://adknowledgeportal.synapse.org/). Omics data of LGG, KIPAN, and BRCA, as well as the grade information of LGG patients, were obtained from The Cancer Genome Atlas Program (TCGA) through Broad GDAC Firehose (https://gdac.broadinstitute.org/). PAM50 breast cancer subtypes of TCGA BRCA patients were obtained through the TCGAbiolinks R package (v2.12.6, http://bioconductor.org/packages/release/bioc/html/TCGAbiolinks.html).

Field-specific reporting

Please select the one below that is the best fit for your research. If you are not sure, read the appropriate sections before making your selection.

[x] Life sciences [ ] Behavioural & social sciences [ ] Ecological, evolutionary & environmental sciences

For a reference copy of the document with all sections, see nature.com/documents/nr-reporting-summary-flat.pdf

Life sciences study design

All studies must disclose on these points even when the disclosure is negative.

Sample size	The sample sizes for the datasets were based on the publicly available datasets, which are selected to have sufficient samples for training and testing.
Data exclusions	No data were excluded from analyses.
Replication	Since this is a computational algorithm, multiple datasets were tested.
Randomization	Sample randomization was based on the public datasets used in this study, we did not apply additional randomization.
Blinding	The tests did not specifically select samples for each group, samples in the training and testing process were randomly selected.

Reporting for specific materials, systems and methods

We require information from authors about some types of materials, experimental systems and methods used in many studies. Here, indicate whether each material, system or method listed is relevant to your study. If you are not sure if a list item applies to your research, read the appropriate section before selecting a response.

Materials & experimental systems

n/a	Involved in the study
[x]	[ ] Antibodies
[x]	[ ] Eukaryotic cell lines
[x]	[ ] Palaeontology and archaeology
[x]	[ ] Animals and other organisms
[x]	[ ] Human research participants
[x]	[ ] Clinical data
[x]	[ ] Dual use research of concern

Methods

n/a	Involved in the study
[x]	[ ] ChIP-seq
[x]	[ ] Flow cytometry
[x]	[ ] MRI-based neuroimaging
